# Synthesizing a pragmatic and systemized measure of universal health coverage: verifying the circumstances of mortality CATegories of death investigated by verbal autopsy

**DOI:** 10.3389/fpubh.2025.1422248

**Published:** 2025-04-03

**Authors:** Ningxin Zhu, Abdul Wahab, Mária Bartušová, Nawi Ng, Laith Hussain-Alkhateeb

**Affiliations:** ^1^Global Public Health Research Group, School of Public Health and Community Medicine, Institute of Medicine, Sahlgrenska Academy, Gothenburg University, Gothenburg, Sweden; ^2^Sleman HDSS, Faculty of Medicine, Public Health and Nursing, Universitas Gadjah Mada, Yogyakarta, Indonesia; ^3^Department of Biostatistics, Epidemiology and Population Health, Faculty of Medicine, Public Health and Nursing, Universitas Gadjah Mada, Yogyakarta, Indonesia; ^4^Department of Preventive and Clinical Medicine, Faculty of Public Health, Slovak Medical University, Bratislava, Slovakia; ^5^Population Health Research Section, King Abdullah International Medical Research Center, Riyadh, Saudi Arabia

**Keywords:** verbal autopsy, health system, social determinants, universal healthcare, civil registration and vital statistics, circumstances of mortality CATegories

## Abstract

**Background:**

Vital data on births, deaths, and causes of death are primarily captured by civil registration and vital statistics systems, which are vastly lacking or inadequately operating in resource-constrained settings. Out-of-health facility deaths remain prevalent and often pass without adequate medical certification, leading to gaps in understanding the medical, social, logistical, and health system circumstances contributing to these deaths. Verbal autopsy (VA), as a standardized and scalable method, is well designed to fill gaps by eliciting causes of death information at the population level. Circumstances Of Mortality CATegories (COMCAT) is a newly integrated concept within VA to identify and quantify likely circumstantial factors contributing to deaths, mainly from social and health system aspects. COMCAT, previously tested in South Africa and Saudi Arabia, show potential to systematically categorize circumstances of death at a population scale. This study intends to verify the process of COMCAT by assessing its plausibility and relevance in generating sensible applications in Indonesia.

**Methods:**

InterVA-5, a machine learning-based VA tool, was used for processing and interpreting medical and non-medical (COMCAT) causes of death for data collected between 2017 and 2021 in the Sleman Health and Demographic Surveillance System. Cause-specific mortality fractions and the corresponding COMCAT categories were derived for each cause of death.

**Results:**

Lack of recognition of the illness severity among families attributed mostly to deaths in the Sleman region. The proportions and ranks of each COMCAT were consistent with the known health information of the population in the setting, which speaks to the plausibility of these findings. The novel geo-mapping component of this tool application adds value to monitoring “hot spots” and their social and health system determinants.

**Conclusion:**

Geo-temporal COMCAT information shows sensible applications of the utility of the VA by producing plausible circumstantial information on population mortality in time and space.

## 1 Introduction

Universal health coverage (UHC) is a crucial Sustainable Development Goal (SDG) toward adequate and timely access to healthcare services mainly for the most disadvantaged societies, which remains among the global health unfinished agenda (1). In the context of understanding causes of death statistics, how UHC is measured and compared between and within countries is important for routine monitoring and assessment, but no systematic approach currently exists (2–5). Globally, over half of the annual deaths pass without medical certifications of their causes, mostly in low- and middle-income countries (LMICs) in Africa and Asia (6, 7). This is typically due to the poor coverage and inadequately functioning civil registration and vital statistics (CRVS) systems, which should count and account for each and every individual and their vital events including birth, death, and cause of death (COD) information in a country (4). Since most deaths in LMICs are community deaths, these deaths are more likely to occur outside healthcare facilities due to several social and health system barriers hindering timely and adequate access to healthcare (8).

Verbal autopsy (VA) is a validated and well-established method for eliciting COD when the medical certification of COD is inadequate, particularly in settings where CRVS is not established (9). VA has two main phases: first, collecting information on the deceased’s most immediate signs and circumstances preceding death using a standardized interview between a trained fieldworker or community officer and the deceased’s close caregiver or relative. Second, the collected data are interpreted to derive probable population-level COD, which follows an abridged form of the international classification of diseases codes (ICD) (9). Until recently, the interpretation of VA data was conducted by physicians; however, given the large number of deaths that lack certifications and the time and cost of the physician review process, automated interpretation using mathematical algorithms has been increasingly used in more recent times (7, 10). In recent decades, many software programs have been developed to automatically interpret VA data, of which the most widely used is InterVA, but SmartVA and InSilicoVA are also commonly used (7, 10, 11). Bayesian probabilistic modeling, which constitutes the InterVA model, is used to interpret VA data by generating likelihoods of the most probable COD for each death, thereby assigning COD to deaths (12, 13). While applied in numerous research settings and routine health data collection across 22 LMICs, the InterVA software has been thoroughly validated as a reliable and consistent tool in assigning medical COD (7, 14–17).

Nevertheless, mere dependency on the medical COD is not sufficient for the measure of UHC, particularly since multiple circumstances can be present in the pathway between illness and death, which can influence the likelihood of deaths to occur (7). Social autopsy (SA) is a further developed method to investigate death, mainly in understanding the circumstantial factors related to deaths, including social, economic, and health system aspects (7, 18, 19). SA has been frequently used with VA in registered populations; nevertheless, SA tends to demand time and cost if used as a sole tool, and unlike VA, it will not be amenable to large-scale applications or automated interpretation (19–22). To facilitate routine and harmonized information about circumstantial factors related to deaths, the Circumstances Of Mortality CATegories (COMCAT) concept was introduced and tested more recently (23, 24). COMCAT is a module that was incorporated into InterVA (version 5), which utilizes a probabilistic approach similar to that applied by InterVA for assigning medical COD. Based on circumstantial information collected from the World Health Organization (WHO) VA standardized questionnaire, the InterVA-5 generates the probability of a circumstance category for each death in the defined population (23, 25). COMCAT is not intended to replace SA but to automatically classify circumstantial factors for deaths at large population scales without requiring additional effort to existing VA routines (23, 25).

In countries where VA is routinely used for population health surveillance, the application of COMCAT has been part of the WHO VA standard and be used by health district managers to capture population-level medical and non-medical (COMCAT) causes attributing to the ultimate deaths. The circumstantial information can conveniently describe the population’s status of accessing health services. Should more specific geo-coded cases be available, the distribution of associated barriers at different geographical levels can be further illustrated. This geo-temporal vital information permits a more systematic and pragmatic measurement of UHC (26). COMCAT can also facilitate a better understanding of the most critical health needs of a defined population, and consequently, appropriate interventions can be introduced. Therefore, it holds promising potential for advancing SDGs associated with cause-specific mortality reduction (Targets 3.1, 3.2, 3.4, 3.6, 3.9) and likely to contribute to equity improvements when integrated with broader systemic interventions (1, 27, 28).

The proof of COMCAT concept was tested using VA data from the Agincourt Health and Socio-Demographic Surveillance System (HDSS), and its plausibility and applicability were more recently assessed using VA data from the Africa Health Research Institute HDSS in South Africa and in the context of mortality related to diabetes mellitus in Saudi Arabia (23–25). The published findings demonstrated adequate plausibility of COMCATs of what would have been expected in those settings. However, more demands for the use of COMCAT have been apparent in new settings coupled with concerns about its validity and practicality that would be associated with its application in cross-settings (17).

Using VA data from Sleman HDSS in Indonesia, this study intends to assess the plausibility and sensible applicability of COMCATs in this Asian context by applying time and geographical assessment. This study has more specific objectives to address (i) how the derived medical and circumstantial COD findings using the VA-COMCAT system are being consistent with what might be expected in this targeted population? and (ii) how relevant and applicable the geo-temporal information provided by the COMCAT system is for local health decision-making and health system development in the Sleman district and in Indonesia?

## 2 Materials and methods

### 2.1 Study setting and population

Indonesia is a lower-middle-income country with a population of 273 million, which includes various ethnic and cultural groups with an average life expectancy of 72 years based on 2020 reports (29, 30). As per the epidemiological transition, non-communicable diseases (NCDs) are becoming increasingly prevalent; however, communicable diseases and maternal, prenatal, and nutrition conditions caused 19% of deaths in 2019 (29, 31). Although advancements in the health system have led to significant improvements in the overall health of the Indonesian population in recent decades, there are still crucial challenges in health disparities between rural and urban areas (30).

The Sleman HDSS was established in Sleman District, Yogyakarta Special Region, Indonesia, in 2015. Sleman District is predominantly urban, with higher levels of education, life expectancy, and per capita healthcare costs compared to the national average (32). In the Sleman HDSS, representative samples were taken from the entire population in the Sleman district (33). Data were collected from the baseline (Wave 1 data collection in 2015) and the subsequent annual data collection of vital events (including VA data). A published study has described the full steps of sampling and each variable in detail (32). The WHO 2012 VA standard has been used since the first wave of data collection in Sleman HDSS, which permits the application of COMCAT since it was introduced in the WHO VA questionnaire in 2012 (32).

This study used data from death cases recorded in wave 3 (2017) to wave 7(2021) data collection in the Sleman HDSS in Indonesia. Due to the comprehensive annual population-level data in the Sleman HDSS on population transition, health status, social context, and its variant settings in terms of geographical location, economic level, and urban and rural distribution, data from Sleman HDSS would be an appropriate and relevant source to verify the applications of COMCAT (23).

### 2.2 Collection and interpretation of VA data

VA data were sought for all deaths spanning the period 2016 to 2021 using standardized WHO VA interviews between a trained fieldworker and the deceased’s close caregiver during registered annual home visits (9, 32). Collected VA data were processed for each death case using the InterVA-5 tool, which generates up to three most probable medical COD and their likelihood of attributing to the specific COD (13). For death cases collected in wave 6 and wave 7 during the corona virus disease 2019 (COVID-19) pandemic, the VA questionnaire included 10 additional questions related to COVID-19, and based on the corresponding responses, the possibility of dying from COVID-19 was also calculated. As an emergent ICD, COD attributed to COVID-19 was calculated separately from the other causes since at the time there was no standard statistical protocol within InterVA-5 to combine the COVID-10 likelihood with other causes. To incorporate COVID-19 and its likelihood into the data analysis alongside the three most probable COD and their likelihoods, we retained the likelihood of COVID-19 and took the likelihood of the other three COD and multiplied it by the likelihood of not dying from COVID-19 (obtained as 1 minus the likelihood of COVID-19). The resulting values represented the likelihood of these three COD, which, combined with the likelihood of COVID-19, yielded the probabilities of COVID-19 and the three other most likely COD, ensuring that the overall likelihood proportions summed up to 100%.

InterVA was used because it has been extensively validated over the past 20 years and is currently the only automated software that can assign COMCAT categories to deaths (13). InterVA is the most widely used open-source model for automated interpretation of VA data and can be freely accessed at www.interva.net (13). The 5^th^ version of InterVA can process WHO VA 2012 and WHO VA 2016 standard data, both of which include previous VA questions about medical COD and 10 newly added questions related to social and health system circumstances (13, 34). It can also process responses to the 10 questions about COVID-19 (35).

In the process of yielding and interpreting COMCAT using InterVA-5, the likelihood of each COMCAT was calculated separately by the COMCAT sub-model developed in InterVA-5, which processed all indicators derived from the 10 questions on social and health system provided under the WHO VA 2016 standard (23, 34). There are seven predefined categories in COMCAT system based on their social common practices, behaviors of individuals, logistical barriers, and the responsiveness of the health system toward the individuals’ health emergencies and needs: Traditions, Emergencies, Recognitions, Resources, Health systems, Inevitability, and Multiple (Table 1). The likelihood of each COMCAT is calculated using a Bayesian probabilistic sub-model, similar to the one used to estimate the medical COD under InterVA model—that Bayes theorem combined with a set of prior probabilities linking input indicators to medical and non-medical COD (including the 10 circumstantial questions) to estimate probability of each cause (12, 13). The sum of definitive six COMCAT’s likelihoods is 100%. If the likelihood of one of the first six COMCATs exceeds 50%, that category is assigned to the case. In occasions where no case could reach 50% for any of the six COMCATs, the ‘multiple’ category applies. After these processes, each death case had been assigned three medical COD (four COD including COVID-19 for death cases collected in wave 6 and wave 7) and their likelihood and one COMCAT.

**Table 1:** Summary of all seven circumstances of mortality categories (COMCAT) and their definitions (23).

COMCAT	Definitions
Traditions	Traditional practices or beliefs influenced health-seeking behavior and the pathway to death
Emergencies	Sudden, urgent, or unexpected conditions leading to death, which probably precluded life-saving actions
Recognition	Lack of recognition or awareness of serious disease (e.g., symptoms or severity) negatively influenced health-seeking behavior
Resources	Inability to mobilize and use resources (e.g., material, transport, and finances) hindered access to healthcare
Health systems	Problems in getting healthcare/ treatment despite accessing health facilities (e.g., related to admissions, treatments, and medications)
Inevitability	Death occurred in circumstances that could not reasonably have been averted (e.g., very older adult or recognized terminal conditions)
Multiple	A combination of the above categories affected the pathway to death; no single factor predominated

### 2.3 Data analysis

After assigning COD and COMCAT classification to each death case, those VA data were consolidated at the population level as VA is purposively designed for reviewing population-level profile of death. Meanwhile, descriptive statistics were employed after reclassifying the medical COD into their respective corresponding disease types, and VA data at population level were presented by different years and across different districts to further discuss the plausibility of the data produced by COMCAT.

Findings from VA analysis are derived and interpreted as population-level measures of COD and COMCATs categories and represented by the cause-specific mortality fractions (CSMFs) for each COD and COMCAT. The seven COMCAT categories were then ranked across all major COD categories using the average value of the derived probabilities for the corresponding COMCAT. We then aggregated the rankings and discussed their plausibility in correspondence with the results of disease-specific studies conducted in the Sleman region, i.e., whether the rankings are consistent with what might be expected in this targeted population.

In addition, the mortality data (including medical COD and COMCATs) were stratified in time, age, and geographical area. The results of the data stratified by time are presented as stacked area charts to better show the proportions of different COD and COMCATs across all deaths each year. For assessing the derived results by some background and characteristics factors, we categorized all cases into five age groups along with the corresponding proportions of COMCATs and CODs and presented them as stacked column charts. We compared these two types of proportions and then discussed them in relation to information on population health and health systems in different age groups in Sleman District and Indonesia. For the spatial assessment, we calculated the proportion of COD and COMCATs among all deaths across 17 sub-districts and discussed both medical and non-medical measures in relation to the relevant statistics for each sub-district on the Sleman HDSS website (36).

## 3 Results

During the data collection of waves 3 to 7, a total of 979 deaths were recorded between 2016 and 2021 and subsequently followed by VA interviews within the Sleman HDSS. For medical COD, more than half of all deaths (54.3%) were attributed to NCDs, with stroke accounting for 15.1% of all deaths, making it the leading COD. Approximately 21.2% of deaths were attributed to infections, with COVID-19 accounting for 8.6% of all deaths, mostly clustered in 2021. Approximately 5.1% of deaths were due to external causes (of which 2.0% were to road traffic accidents, 2.4% were to other transport accidents), 1.5% were to pregnancy- and neonatal-related causes, and 18.0% were indeterminate. The derived COMCAT probabilities revealed that 36.7% of all cases were assigned to ‘recognition’, 35.1% to deaths due to terminal conditions (inevitable), 11.4% to ‘Emergencies’, 6.2% to ‘Resources’, 5.5% to ‘Health Systems’, 2.2% to ‘Traditions’, and 2.8% to ‘Multiple’.

**Figure 1:**
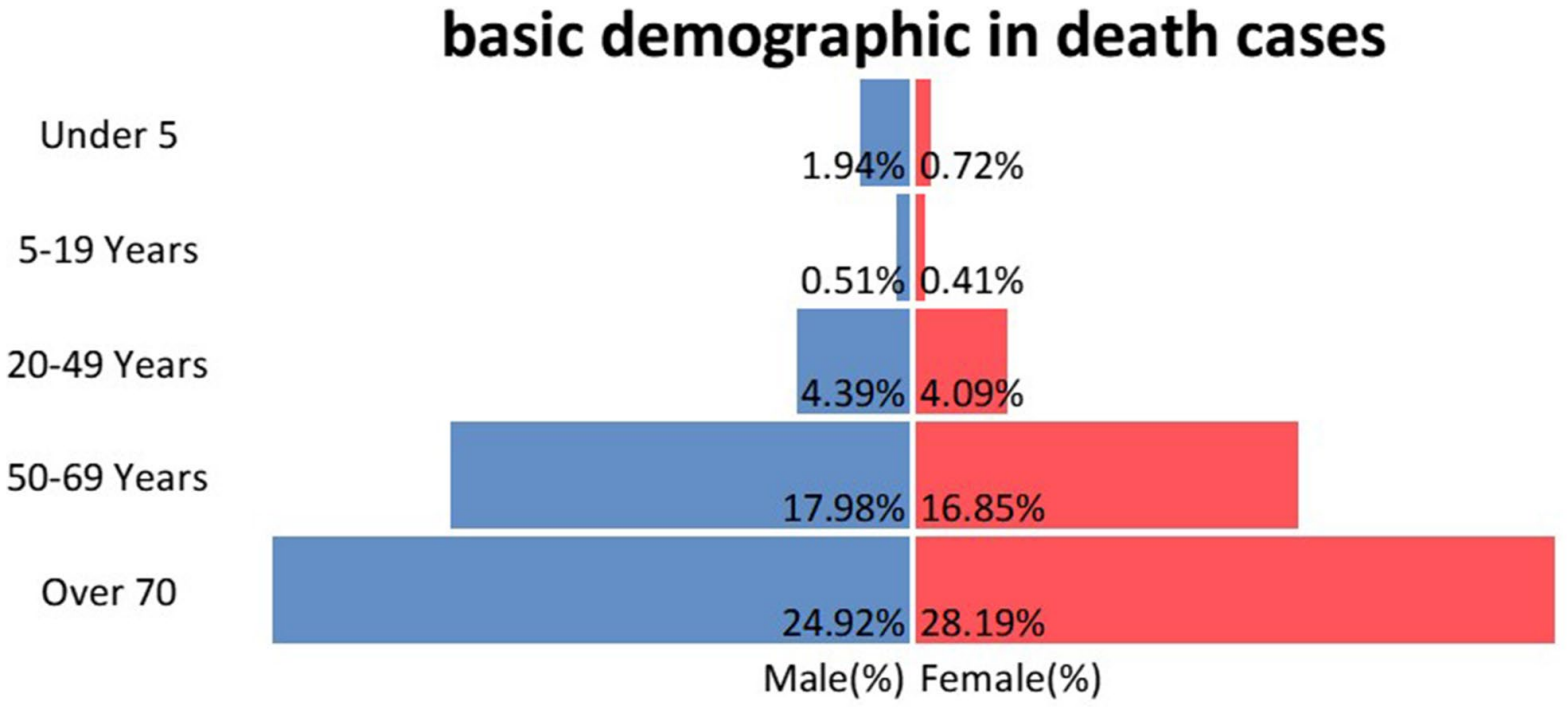
Percentage of death cases by age group and gender.

At the targeted population, most deaths occur after the age of 50 years old, with the majority being among the >70 years old group (34.8% among 50–69 and 53.1% among over 70). In overall, the mortality was almost identically distributed by sex (49.7% among males; Figure 1).

**Figure 2:**
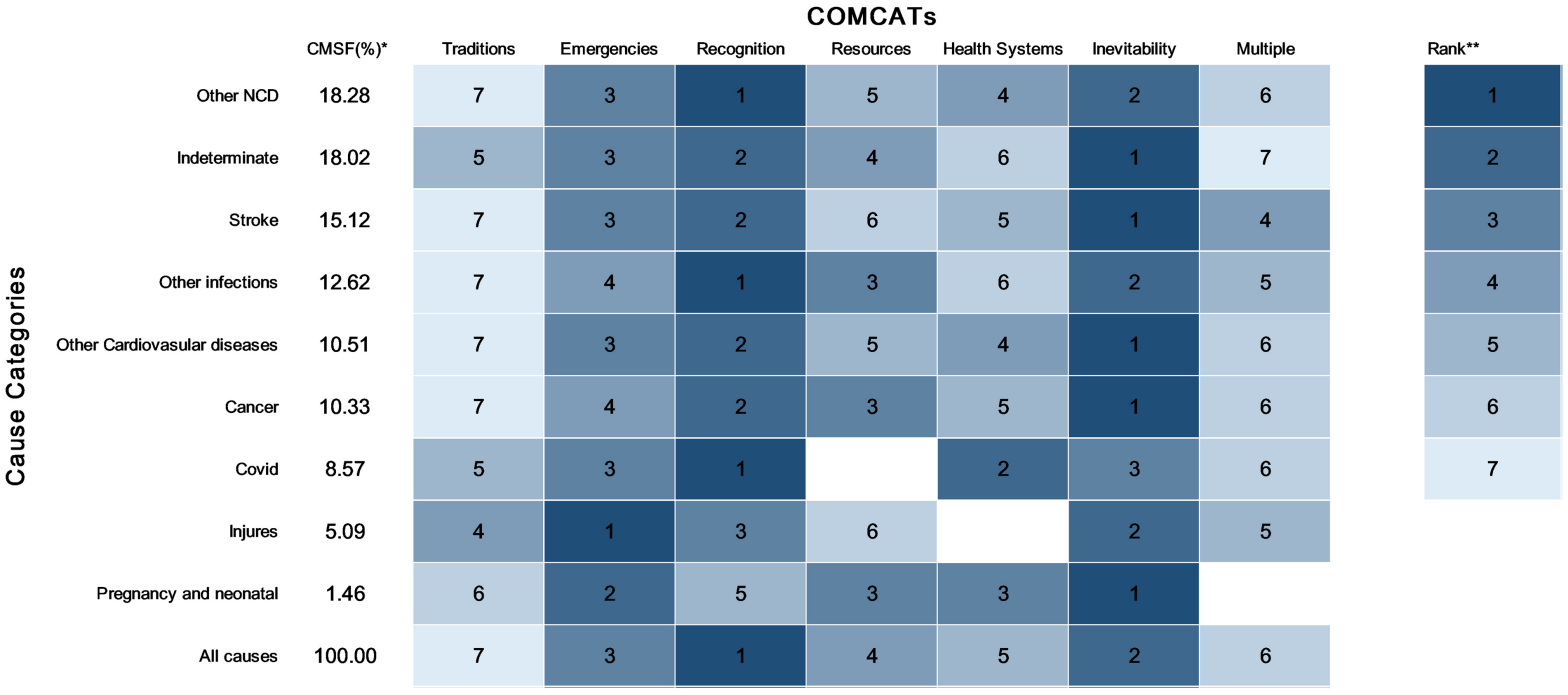
COMCATs ranked within each major cause category for 979 deaths in the Sleman HDSS. *Cause-specific mortality fractions for each major cause of death (COD) category (e.g., 15.1 means that stroke caused 15.1% of all deaths). **Rank of the proportion of each COMCAT within deaths caused by each COD (1—highest proportion and 7—lowest proportion), darker blue color indicates the higher (rank) contribution of the corresponding COMCAT to the causes of death.

Figure 2 shows the ranking of the relevant COMCATs across major medical cause categories. For most main medical causes, ‘Inevitability’ was the most dominant COMCAT except for infections, NCDs except cancer, stroke, and other CVDs and injuries, which are mostly associated with ‘recognition’ and ‘emergencies’. In contrast, ‘tradition’ was ranked low for almost all major COD. NCDs, such as stroke, cardiovascular diseases, and cancer, were closely correlated with ‘recognition’. The lack of recognition was also most relevant to infections including COVID-19 according to the COMCAT analysis. However, other COMCATs are ranked differently between ‘COVID-19’ and ‘other infections’, with the ‘health system’ ranking second only to ‘recognition’ in ‘COVID-19’, while in ‘other infections’ the ‘health system’ is ranked fifth.

**Figure 3:**
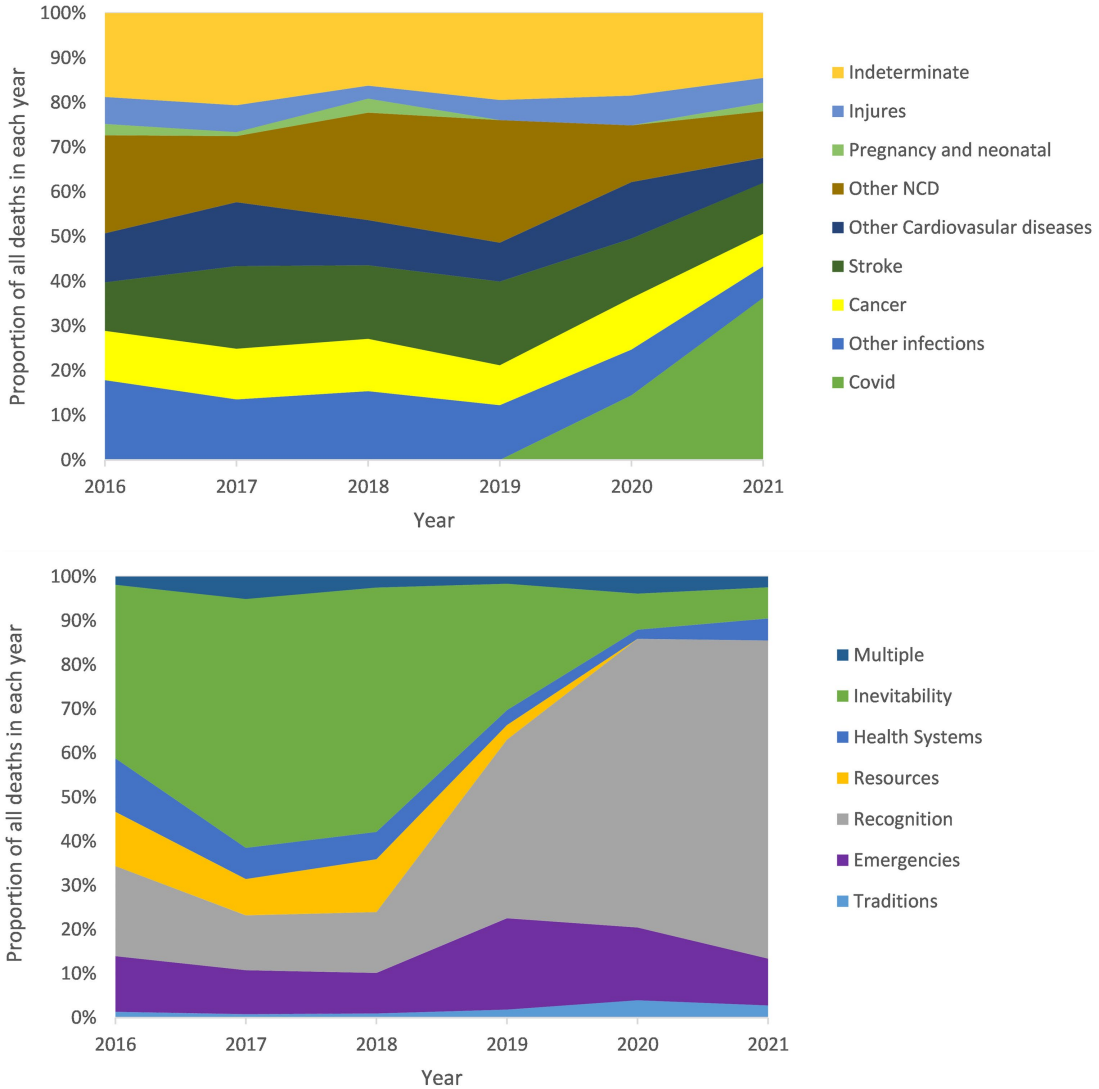
Proportion of each COD and COMCAT to annual deaths by year in the Sleman HDSS.

Figure 3 demonstrates the changes in the proportion of deaths counted per year for each COMCAT and major medical causes over time between 2016 and 2021. Before the year 2019, NCDs, particularly cardiovascular diseases including stroke, accounted for the main proportion of annual deaths; however, after 2019, it has been gradually taken over by COVID-19. The proportions of injuries, pregnancy, and neonatal causes and indeterminate have not changed significantly in this 6-year period, with pregnancy and neonatal causes always accounting for the least death cases. In parallel for COMCAT, ‘inevitability’ has been dominating before 2019, followed by a decrease in the proportion with an increasing proportion of ‘recognition’. In addition, the proportion of ‘health systems’ decreased until 2020, and the proportion of ‘resources’ increased in 2018.

**Figure 4:**
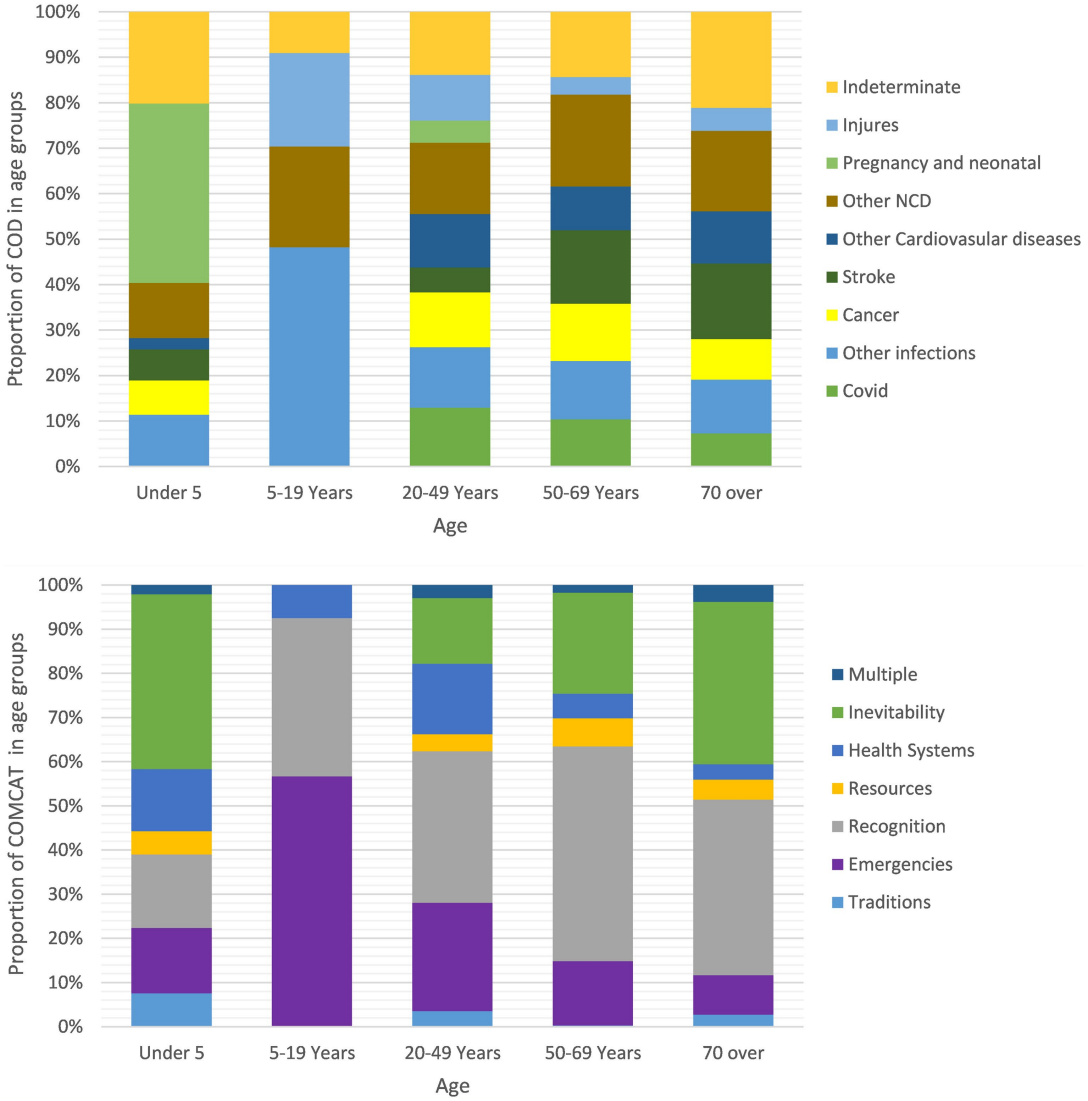
Proportions of COD categories and COMCATs within 5 age groups, the Sleman HDSS 2016–2021.

Figure 4 demonstrates the proportions of all deaths across the 5 age groups in terms of classifications of major medical COD and COMCATs. In the age group of 5–19 years, the proportion of ‘injuries’ is high compared with other age groups, concordance to ‘emergencies’ scoring highest for COMCAT. The proportion of ‘inevitability’ is high in the under 5 age and across the three adult groups, and the older the group the higher the proportion of it.

**Figure 5:**
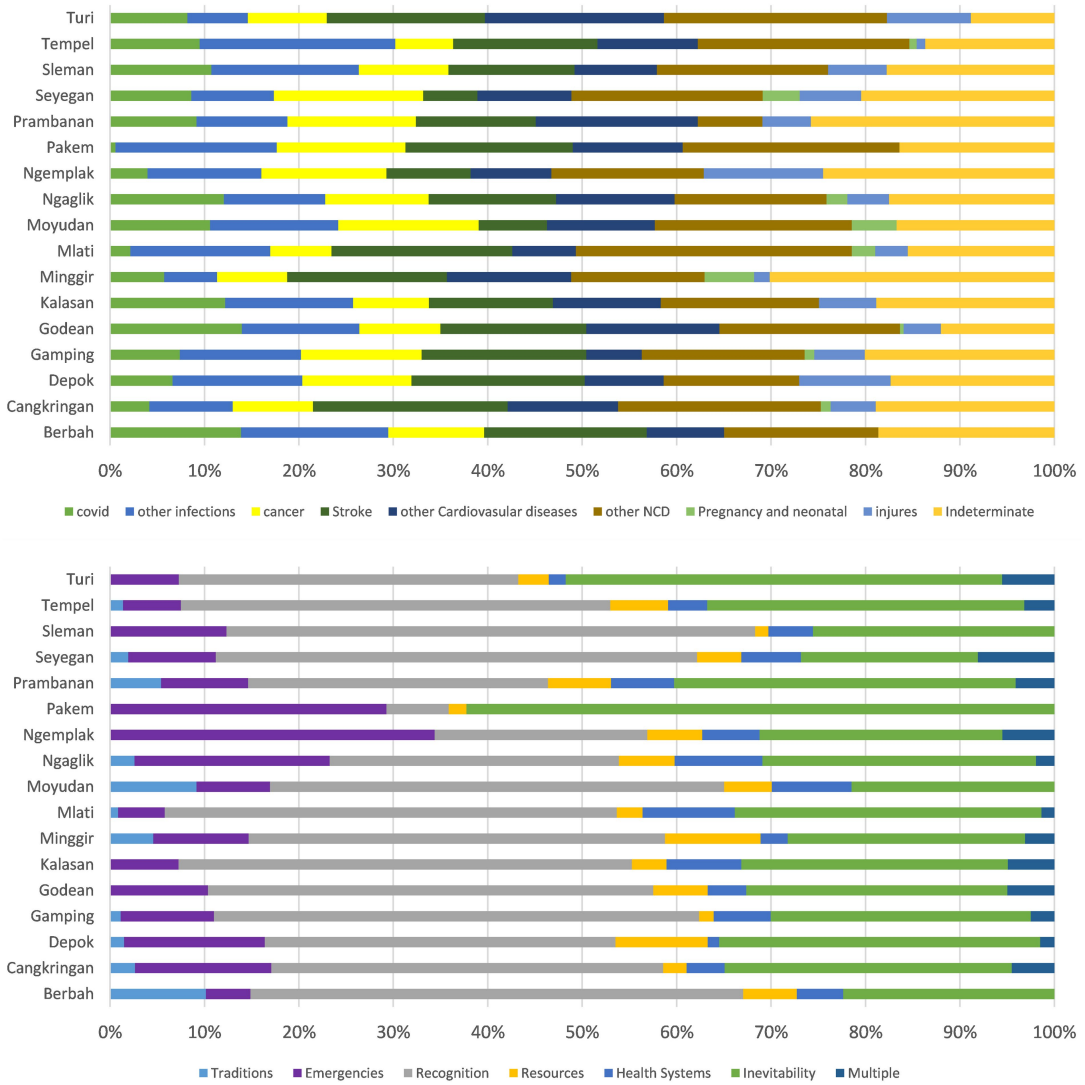
Proportions of COD categories and COMCATs within 17 subdistricts, the Sleman HDSS 2016–2021.

Figure 5 shows the proportion of all deaths across 17 subdistricts in Sleman district for both the classification of medical COD and COMCATs. In these subdistricts, Tempel, Berbah, and Minggir have lower proportions of ‘Injuries’, and Tempel and Berbah have a lower proportion of ‘emergencies’, while Minggir has a higher proportion of ‘Pregnancy and neonatal’ compared with other subdistricts. Pakem has a low proportion of ‘COVID-19’, and it also has a low proportion of ‘Recognition’ in terms of COMCAT.

## 4 Discussion

The prime aim of the COMCAT system is to provide local and national health authorities with a standardized assessment of social and health system factors contributing to the population’s deaths such as issues related to the adequacy of emergencies services, problems with recognizing disease severity, decision between pharmaceutical and traditional medicine, and availability of resources for mobilizing to seek care and problems with admission, treatment, and care at health facilities. COMCAT can arguably be used for recognizing such key modifiable social, logistical, and perceived health system factors attributing to the ultimate deaths in a society, leading to timely and adequate directions of health policies and public health interventional programs. For instance, the current study revealed that stroke is the leading COD group, which corresponds to the ‘recognition’ category from the COMCAT assessment, suggesting that more adequate health promotion and educational programs are warranted for this population. In addition, deaths at younger ages in Sleman HDSS are often associated with injuries and infections, according to this study, while deaths of older ages are linked to NCDs, with their corresponding COMCATs categorized as ‘emergency’ and ‘inevitability’. This assessment suggests that while older individuals are more likely to access hospitals and die there—since inevitability is associated with recognized terminal illnesses often under medical supervision—health system aspects related to emergency services remain underdeveloped.

### 4.1 Plausibility of COMCAT

In this section, we present and discuss our interpretations of the plausibility of the COMCAT. The study period coincided with the COVID-19 pandemic, which attributed to unprofiled deaths among medical practices. At the early phase of COVID-19—which overlaps with the interval of this study—lay people lacked basic awareness of the COVID-19 manifestations and its natural history leading to increased challenges associated with ‘recognizing’ the disease symptoms, which is what the COMCAT model also recognized as top rank category. Particularly for an emergent COVID-19 pandemic, it was apparent that the majority of the population were not well informed about its symptoms but were presumably only aware of the preventive measures, which can largely influence the severity of the disease at the time of medical consultation (37, 38). Nevertheless, the medical services (health system) were technically overwhelmed by the influx of patients coinciding with the early time of the pandemic and coupled with imposed restrictions on people’s mobility, collectively leading to limited access to healthcare facilities. This assessment plausibly aligned with COMCAT, which suggested problems accessing “health system” to come second after issues related to “recognition.” In addition, those who accessed and died at hospitals were severe and terminal-stage cases as declared by COMCAT suggesting “inevitability” to come third in the rank.

An overall higher probability of ‘inevitability’ was derived from the Sleman HDSS COMCAT assessment. This finding can possibly be attributed to the fact that more than half of the death cases were NCD-related. Such diseases tend to have a higher hospitalization rate and probably a better chance of receiving appropriate treatment if when hospitalized. In occasions of inevitable deaths despite receiving in-hospital care, families would have clearly declared this circumstance due to their direct exposure to medical opinions. Traditional treatments are often sought as physical treatments that are not life-threatening by wealthy groups in Indonesia and who usually enjoy good access to professional healthcare (39, 40). It is therefore reasonable that ‘tradition’ as proposed by COMCAT attributed to only a few deaths.

Based on the ranking illustrated by Figure 2, COMCAT assignment across all medical cause categories appears plausible from a medical and public health point of view. For instance, injuries are more correlated with ‘emergencies’, a relationship that makes sense given the fact that most of the cases in the ‘injuries’ group were caused by transport accidents, and they tend to occur very rapidly leading to progressive emergency situations. This may reflect the deficiencies in the provision of emergency medical services, particularly ambulance services in Indonesia, which is also declared in other published reports (41, 42). In addition to COVID-19-related deaths, stroke, cardiovascular diseases, and cancer are strongly associated with ‘recognition’ according to COMCAT. This makes sense as these particular NCDs are usually chronic onsets, with detectable signs and symptoms only appearing when they are more severe, and therefore can easily be ignored leading to delayed medical attention (43). A study on caregivers of stroke patients in Indonesia noted that many caregivers do not have adequate stroke-related expertise and that the status of domestic workers as informal caregivers is common in Indonesia (44). Such cross-sectional findings were also consistent with information provided by COMCAT. The lack of ‘recognition’ of the severity of illness was also most relevant for infections according to the COMCAT analysis, causing delayed diagnosis and intervention for infectious diseases (45). This is plausibly correct considering that most infectious diseases profiling the death incidences in this setting have overlapping symptoms, which requires effective early diagnosis and treatment based on symptoms in responding to infectious diseases. In addition, the ‘health system’ ranked second only to ‘recognition’ category in ‘COVID-19’, while in ‘other infections’ the ‘health system’ category is ranked 5th. This difference is sensible in view of the lack of effective treatment for COVID-19 where certain groups of patients, mainly those most vulnerable or comorbid can still die despite timely access to healthcare. This scenario can plausibly attribute ‘health system’ factors to these death groups.

According to Figure 3, between 2019 and 2021, the proportion of ‘recognition’ increased with a decrease in the ‘inevitability’, and the same trend occurred for COVID-19 and NCDs. Possible reasons for these observations can be that many NCDs have been shown to exacerbate COVID-19, making it more likely to cause death, and that in such cases, death is often attributed to COVID-19 (46, 47). Furthermore, during the COVID-19 pandemic, patients with NCDs are less likely to be hospitalized compared to pre-COVID time due to the urgent healthcare need for patients with COVID-19 and the restriction policies during the pandemic. This can result in patients not receiving the medical care needed or direct assessment of medical expertise and thus reducing the ‘inevitability’ likelihood associated with these deaths. In 2018, the percentage of ‘resources’ increased slightly compared to the previous year. This trend can be attributed to the 2018 closing-down of an easy-to-reach hospital for residents in Sleman (48). According to the official local statistics of Sleman, the medical facilities of the medical institutions in Sleman were becoming better equipped during the record time, and the number of professional medical workers was increasing on a yearly basis, which can reasonably explain the decrease in the percentage of the health system COMCAT over the years until the outbreak of COVID-19 (48).

As illustrated in Figure 4, the distribution of COD and COMCAT across age groups seems sensible. For deaths among the 5–19 years age group, the proportion of ‘injuries’ is relatively high, which can correlate with the higher proportion of ‘emergencies’ in this age group. The distribution of ‘inevitability’ across age groups is also reasonable, and the older the group the higher the proportion of ‘inevitability’, except for the under-5 age group. Figure 5 shows the proportions of COMCAT and COD across 17 subdistricts in the Sleman district. Tempel and Berbah have lower proportions of ‘emergencies’, which can be reasonably explained by the fact that deaths due to injuries occur less in these two subdistricts, as observed from the COD figure. The opposite is true for Ngemplak, which has a high proportion of both ‘emergencies’ and ‘injuries’. In Pakem, the proportion of ‘recognitions’ is much lower than in other subdistricts, which corresponds to the low proportion of ‘COVID-19’.

### 4.2 The applicability of COMCAT

From a public health perspective, the application of COMCATs has the potential to add value to the use of VA data by enhancing health policymaking in Indonesia. Until recently, the VA relied solely on CSMF as a population-level measurement of CODs for describing the epidemiology of the burden of diseases in a defined population. Such mortality data can provide a more detailed picture of the health problems that exist in a population. However, simply recognizing existing burdens does not solely support health decision-making without a thorough understanding of what types of interventions are particularly needed to alleviate health problems. For example, the CMSF data describe diseases accounting for a high proportion of all deaths. By combining COMCATs with the CMSF data to obtain a ranking of circumstantial factors within the diseases (e.g., Figure 2), public health epidemiologists can accordingly use this augmented piece of information for timely allocation of circumstantial factors that may have a wider impact on the health status of the population and accordingly design more adequate interventions based on those circumstantial factors. Routine use of VA and COMCAT while integrating temporal and geographical information of death (as shown in Figure 4) can collectively facilitate additional comprehensive monitoring dashboards for detecting hot spots of urgent resource allocations and relevant interventional programs.

In Indonesia, health disparities between populations are rapidly developing and those living in poorer areas are of major health issues (30). People in poorer areas tend to have a higher chance of dying outside a healthcare facility, and information on out-of-hospital deaths is either incomplete or inconsistent compared to deaths at health facilities. These unequal deaths in relation to vulnerable societies in a population create gaps in health equities. The collection of VA data can lead to better documentation of community deaths and its application as a combined VA-COMCAT tool. The Indonesian government has paid closer attention to the burden of the health of the most disadvantaged population, with increased efforts to promote a universal health insurance system since the early 21st century (30). The data obtained from COMCAT address populations without complete health record data and can help managers make better use of government funding and thus move closer to the goal of health equity. Nationally, there is a health information system called KOMDAT in Indonesia, which collects health records based on data uploaded by the district health department. Data from the district are compiled from data reported by local health facilities, and the national health insurance agency (BPJS) also has a system collecting patients’ health data (49). Both systems only contain data reported by health facilities, which can lead to ignoring health information from outside facilities. This lack of data recording can result in similar needs being overlooked and thus no effective intervention for these needs for healthcare services being presented. In this case, data from COMCATs can complement this information. According to the WHO report, death information recording is at a low capacity in Indonesia (50). Hence, systematic applications of such standardized VA and COMCATs process can enrich this gap.

COMCATs would be helpful to Indonesian population health, particularly during emergency situations such as the COVID-19 outbreak or other climate-sensitive disease outbreaks such as dengue, where their distributions are not normally distributed but inextricably linked to most disadvantaged communities (51). VA has empirically shown to be reliable in determining whether a death is due to COVID-19, and standardized use of VA and SA in humanitarian crises can be effective in providing additional information on population deaths during pandemics (52, 53). The utility of COMCAT can provide similar information as that provided by SA while also avoiding the duplication of lengthy interviews that often occurs in SA during data collection (7, 20, 21). Therefore, the simultaneous use of COMCATs in this context will provide a useful spectrum of the social and health system factors associated with local burden of diseases and related risk factors. Combining this with VA data such as age and region will allow managers to better identify vulnerable populations who are more at risk of death from specific diseases and their urgent needs to avoid preventable deaths.

Building on the South African study, which examined the application of COMCATs, our study applies COMCATs to data from an Indonesian population and demonstrates its plausibility and application in this setting. Findings from previous COMCAT studies such as the South African and Saudi reports, as well as findings from the current study, show that the COMCAT model seems to produce sensible, locally relevant and able to reveal local health issues that are typically related to the studied settings (23, 24). COMCAT demonstrates significant potential for application across diverse geographic regions and health information systems, offering valuable insights that can enhance local health data and inform decision-making processes (23, 25). Its adaptability across various geographic, demographic, and political contexts underscores its potential for broader global implementation (54, 55). Against the SGDs, COMCAT is likely to be a frontliner application by means of its advanced machine learning approach which utilizes data via standardized WHO VA tool to address crucial global health agenda such as the UHC.

### 4.3 Limitations

The comparison of findings with existing studies may be subject to bias due to heterogeneity in sample selection. Some referenced studies analyzed data from Indonesia’s entire population, whereas the present study sample focuses on Sleman district, a predominantly urban region (85% urban population). This discrepancy is notable given Indonesia’s national urbanization rate of 57% as rural–urban divides may correlate with disparities in infrastructure access, healthcare utilization, and socio-cultural practices. If more relevant findings can be available with a study population in the Sleman district in the future, they could be used as a more accurate reference. (29) In the context of results generalizability, while the Sleman HDSS assigns statistical weight to adjust for the sampling bias, this weight was not possible to use for the VA process since data were categorically aggregated. In addition, the number of death cases in the age group under 5 was relatively low, and this may have contributed to the lack of representativeness of the results related to child deaths in this study (32). To address this shortcoming, COMCATs could be applied to VA data in areas of Indonesia with high child mortality rates to further confirm its reliability when applied to child deaths in future studies. When obtaining the likelihood of dying from COVID-19, the VA is relatively less reliable if deaths occurred among children due to the complexity associated with the disease symptoms in child age, which may have affected the accuracy of our results when classifying all deaths by medical COD. However, as the proportion of cases of child deaths in our study was low, we presume that this will not have an impact on the overall results (52).

## 5 Conclusion

This study aimed to verify the use of the COMCAT model by assessing its plausibility and practical applicability in Indonesia. Accordingly, the COMCAT system seems capable of unpacking social logistical, and health system factors associated with delayed access to health services and delivering time and space piece of strategic information toward more targeted and effective health policymaking. The geo-temporal application of the COMCAT system can broaden the information on avoidable deaths at the health system level and is likely able to provide more comprehensive information for the progress measurement of UHC.

To further investigate the universal applicability of COMCAT and refine its utility for health equity agendas, future research can focus on context-specific validations of the application of COMCAT that address distinct geographic, demographic, and health systemic disparities, potentially extending the current seven COMCATs to account for novel but crucial dimensions such as ‘migration’, ‘occupation’, and ‘climatic’ factors attributing to deaths. Future research can prioritize populations facing more health challenges (e.g., populations facing the burden of infectious diseases or populations with limited access to healthcare). Furthermore, COMCAT can be applied in the same setting for a longer period to guarantee the stability and permanence of output data and provide reference data for a comparison over time to explore trends in population mortality and health and to evaluate COMCAT’s sensitivity to policy-driven changes.

## Data Availability

The original contributions presented in the study are included in the article/supplementary material, further inquiries can be directed to the corresponding authors.

## References

[1] The United Nations. *What are the sustainable development goals?* (2015). Available online at: https://www.undp.org/sustainable-development-goals.

[2] GBD 2019 Universal Health Coverage Collaborators. Measuring universal health coverage based on an index of effective coverage of health services in 204 countries and territories, 1990-2019: a systematic analysis for the global burden of disease study 2019. Lancet. (2020) 396:1250–84. doi: 10.1016/S0140-6736(20)30750-9, PMID: 32861314 PMC7562819

[3] World Health Organization. Tracking universal health coverage: 2017 global monitoring report. Geneva: World Health Organization (2017).

[4] World Bank Group WHO. Global civil registration and vital statistics scaling up investment plan 2015–2024. Washington, DC: World Bank Group WHO (2014).

[5] Groenewald P Azevedo V Daniels J Evans J Boulle A Naledi T. The importance of identified cause-of-death information being available for public health surveillance, actions and research. S Afr Med J. (2015) 105:528–30. doi: 10.7196/SAMJnew.8019, PMID: 26428743

[6] Mikkelsen LD Phillips DEB AbouZahr CM Setel PWP de Savigny DP Lozano RP. A global assessment of civil registration and vital statistics systems: monitoring data quality and progress. Lancet (British edition). (2015) 386:1395–406. doi: 10.1016/S0140-6736(15)60171-4, PMID: 25971218

[7] Thomas LM D'Ambruoso L Balabanova D. Verbal autopsy in health policy and systems: a literature review. BMJ Glob Health. (2018) 3:e000639. doi: 10.1136/bmjgh-2017-000639, PMID: 29736271 PMC5935163

[8] Adair T Rajasekhar M Bo KS Hart J Kwa V Mukut MAA. Where there is no hospital: improving the notification of community deaths. BMC Med. (2020) 18:65. doi: 10.1186/s12916-020-01524-x 32146904 PMC7061465

[9] World Health Organization. Verbal autopsy standards:The 2016 WHO verbal autopsy instrument. Geneva: World Health Organization (2016).

[10] Tunga M Lungo J Chambua J Kateule R. Verbal autopsy models in determining causes of death. Trop Med Int Health. (2021) 26:1560–7. doi: 10.1111/tmi.13678, PMID: 34498340

[11] Nichols EK Byass P Chandramohan D Clark SJ Flaxman AD Jakob R. The WHO 2016 verbal autopsy instrument: an international standard suitable for automated analysis by InterVA, InSilicoVA, and tariff 2.0. PLoS Med. (2018) 15:e1002486. doi: 10.1371/journal.pmed.1002486, PMID: 29320495 PMC5761828

[12] Byass P Chandramohan D Clark SJ D'Ambruoso L Fottrell E Graham WJ. Strengthening standardised interpretation of verbal autopsy data: the new InterVA-4 tool. Glob Health Action. (2012) 5:1–8. doi: 10.3402/gha.v5i0.19281, PMID: 22944365 PMC3433652

[13] Byass P Hussain-Alkhateeb L D'Ambruoso L Clark S Davies J Fottrell E. An integrated approach to processing WHO-2016 verbal autopsy data: the InterVA-5 model. BMC Med. (2019) 17:102. doi: 10.1186/s12916-019-1333-6, PMID: 31146736 PMC6543589

[14] Mapoma CC Munkombwe B Mwango C Bwalya BB Kalindi A Gona NP. Application of verbal autopsy in routine civil registration in Lusaka District of Zambia. BMC Health Serv Res. (2021) 21:408. doi: 10.1186/s12913-021-06427-y, PMID: 33933096 PMC8088624

[15] Adair T Firth S Phyo TPP Bo KS Lopez AD. Monitoring progress with national and subnational health goals by integrating verbal autopsy and medically certified cause of death data. BMJ Glob Health. (2021) 6:387. doi: 10.1136/bmjgh-2021-005387, PMID: 34059494 PMC8169488

[16] Hazard RH Buddhika MPK Hart JD Chowdhury HR Firth S Joshi R. Automated verbal autopsy: from research to routine use in civil registration and vital statistics systems. BMC Med. (2020) 18:60. doi: 10.1186/s12916-020-01520-1, PMID: 32146903 PMC7061477

[17] Aukes AM Arion K Bone JN Li J Vidler M Bellad MB. Causes and circumstances of maternal death: a secondary analysis of the community-level interventions for pre-eclampsia (CLIP) trials cohort. Lancet Glob Health. (2021) 9:e1242–51. doi: 10.1016/S2214-109X(21)00263-1, PMID: 34332699 PMC8370879

[18] John Hopkins Bloomberg School of Public Health. *Verbal autopsy and social autopsy studies (VASA)*. (n.d.). Available online at: https://www.jhsph.edu/research/centers-and-institutes/institute-for-international-programs/current-projects/verbal-autopsy-and-social-autopsy-studies-vasa/.

[19] Kalter HD Salgado R Babille M Koffi AK Black RE. Social autopsy for maternal and child deaths: a comprehensive literature review to examine the concept and the development of the method. Popul Health Metrics. (2011) 9:45. doi: 10.1186/1478-7954-9-45, PMID: 21819605 PMC3160938

[20] Källander K Kadobera D Williams TN Nielsen RT Yevoo L Mutebi A. Social autopsy: INDEPTH network experiences of utility, process, practices, and challenges in investigating causes and contributors to mortality. Popul Health Metrics. (2011) 9:44. doi: 10.1186/1478-7954-9-44, PMID: 21819604 PMC3160937

[21] Moyer CA Johnson C Kaselitz E Aborigo R. Using social autopsy to understand maternal, newborn, and child mortality in low-resource settings: a systematic review of the literature. Glob Health Action. (2017) 10:1413917. doi: 10.1080/16549716.2017.1413917, PMID: 29261449 PMC5757230

[22] Gupta M Kaur M Lakshmi PVM Prinja S Singh T Sirari T. Social autopsy for identifying causes of adult mortality. PLoS One. (2018) 13:e0198172. doi: 10.1371/journal.pone.0198172, PMID: 29851982 PMC5978887

[23] Hussain-Alkhateeb L D'Ambruoso L Tollman S Kahn K Van Der Merwe M Twine R. Enhancing the value of mortality data for health systems: adding circumstances of mortality CATegories (COMCATs) to deaths investigated by verbal autopsy. Glob Health Action. (2019) 12:1680068. doi: 10.1080/16549716.2019.1680068 31648624 PMC6818104

[24] Alyazidi F Shakely D Alyazidi F Alnasser LA Petzold M Hussain-Alkhateeb L. Social and health system barriers: investigating circumstances of mortality categories (COMCATs) for deceased patients with T2DM in the sub-national Saudi Arabia register. PLoS One. (2024) 19:e0313956. doi: 10.1371/journal.pone.0313956, PMID: 39570846 PMC11581326

[25] D'Ambruoso L Price J Cowan E Goosen G Fottrell E Herbst K. Refining circumstances of mortality categories (COMCAT): a verbal autopsy model connecting circumstances of deaths with outcomes for public health decision-making. Glob Health Action. (2021) 14:2000091. doi: 10.1080/16549716.2021.2000091, PMID: 35377291 PMC8986216

[26] The World Bank. *Universal health coverage:quality, affordable health care is the foundation for individuals to lead productive and fulfilling lives and for countries to have strong economies*. (2022). Available online at: https://www.worldbank.org/en/topic/universalhealthcoverage.

[27] Suthar AB Khalifa A Yin S Wenz K Ma Fat D Mills SL. Evaluation of approaches to strengthen civil registration and vital statistics systems: a systematic review and synthesis of policies in 25 countries. PLoS Med. (2019) 16:e1002929. doi: 10.1371/journal.pmed.1002929, PMID: 31560684 PMC6764661

[28] United Nations. *Ensure healthy lives and promote well-being for all at all ages*. Available online at: https://sdgs.un.org/goals/goal3.

[29] Indonesia. (2022). Available online at: https://data.worldbank.org/country/ID (Accessed April 4, 2022).

[30] Mahendradhata Y Trisnantoro L Listyadewi S Soewondo P Marthias T Harimurti P. *The Republic of Indonesia health system review*. (2017).

[31] World Health Organization. *Global health estimates: leading causes of death*. (2020). Available online at: https://www.who.int/data/gho/data/themes/mortality-and-global-health-estimates/ghe-leading-causes-of-death.

[32] Dewi FST Choiriyyah I Indriyani C Wahab A Lazuardi L Nugroho A. Designing and collecting data for a longitudinal study: the Sleman health and demographic surveillance system (HDSS). Scand J Public Health. (2018) 46:704–10. doi: 10.1177/1403494817717557, PMID: 28752803

[33] Sleman Health and Demographic Surveillance System. (n.d.). Available online at: https://hdss.fk.ugm.ac.id/en/home-en/.

[34] D’Ambruoso L Kahn K Wagner RG Twine R Spies B Van der Merwe M. Moving from medical to health systems classifications of deaths: extending verbal autopsy to collect information on the circumstances of mortality. Glob Health Res Policy. (2016) 1:2. doi: 10.1186/s41256-016-0002-y, PMID: 29202052 PMC5675065

[35] Byass P. *COVID-19 rapid mortality surveillance (CRMS) products*. Available online at: http://www.byass.uk/interva/crms.

[36] Sleman Health and Demographic Surveillance System. *Data visualisation*. Available online at: https://hdss.fk.ugm.ac.id/en/data-visualisation/.

[37] Zhang C Liao WF Ma YM Liang CY. Research on older people’s health information search behavior based on risk perception in social networks-a case study in China during COVID-19. Front Public Health. (2022) 10:946742. doi: 10.3389/fpubh.2022.946742, PMID: 36033751 PMC9400025

[38] Yıldırım M Güler A. COVID-19 severity, self-efficacy, knowledge, preventive behaviors, and mental health in Turkey. Death Stud. (2022) 46:979–86. doi: 10.1080/07481187.2020.1793434, PMID: 32673183

[39] Nurhayati N Widowati L. The use of traditional health care among Indonesian family. Health Sci J Indones. (2017) 8:5600. doi: 10.22435/hsji.v8i1.5600, PMID: 39477629

[40] Peltzer K Pengpid S. Traditional health practitioners in Indonesia: their profile, practice and treatment characteristics. Complement Med Res. (2019) 26:93–100. doi: 10.1159/000494457, PMID: 30572336

[41] Yusvirazi L Ramlan AAW Hou PC. State of emergency medicine in Indonesia. Emerg Med Australas. (2018) 30:820–6. doi: 10.1111/1742-6723.13183, PMID: 30253444

[42] Brice SN Boutilier JJ Gartner D Harper P Knight V Lloyd J. Emergency services utilization in Jakarta (Indonesia): a cross-sectional study of patients attending hospital emergency departments. BMC Health Serv Res. (2022) 22:639. doi: 10.1186/s12913-022-08061-8, PMID: 35562823 PMC9103083

[43] Venketasubramanian N Yudiarto FL Tugasworo D. Stroke burden and stroke Services in Indonesia. Cerebrovasc Dis Extra. (2022) 12:53–7. doi: 10.1159/000524161, PMID: 35313314 PMC9149342

[44] Muhrodji P Wicaksono HDA Satiti S Trisnantoro L Setyopranoto I Vidyanti AN. Roles and problems of stroke caregivers: a qualitative study in Yogyakarta, Indonesia. F1000Res. (2021) 10:380. doi: 10.12688/f1000research.52135.2, PMID: 35186263 PMC8822138

[45] Adli I Widyahening IS Lazarus G Phowira J Baihaqi LA Ariffandi B. Knowledge, attitude, and practice related to the COVID-19 pandemic among undergraduate medical students in Indonesia: a nationwide cross-sectional study. PLoS One. (2022) 17:e0262827. doi: 10.1371/journal.pone.0262827, PMID: 35061848 PMC8782366

[46] Fauci AS Lane HC Redfield RR. Covid-19 - navigating the uncharted. N Engl J Med. (2020) 382:1268–9. doi: 10.1056/NEJMe2002387, PMID: 32109011 PMC7121221

[47] Ghilari YED Iskandar A Wiratama BS Hartopo AB. Joint effect of diabetes mellitus and hypertension on COVID-19 in-hospital mortality stratified by age group and other comorbidities: a cohort retrospective study using hospital-based data in Sleman, Yogyakarta. Healthcare (Basel). (2022) 10:103. doi: 10.3390/healthcare10102103, PMID: 36292550 PMC9601841

[48] Sleman, BPSK. *Sleman County regional statistics in 2019 (Statistik Daerah Kabupaten Sleman Dalam Angka 2019)*. In: Badan Pusat Statistik Kabupaten Sleman. ©BPS Kabupaten Sleman. p. 40. (2019).

[49] Braa J Sahay S Lewis J Senyoni W. *Health information Systems in Indonesia: Understanding and addressing complexity*. (2017).

[50] World Health Organization. Score assessment SUMMARY – Indonesia. Geneva: World Health Organization (2021).

[51] Setiati S Azwar MK. COVID-19 and Indonesia. Acta Med Indones. (2020) 52:84–9. Available at: https://www.actamedindones.org/index.php/ijim/article/view/1426 PMID: 32291377

[52] Duarte-Neto AN Marinho MF Barroso LP Saldiva de Andre CD da Silva LFF Dolhnikoff M. Rapid mortality surveillance of COVID-19 using verbal autopsy. Int J Public Health. (2021) 66:1604249. doi: 10.3389/ijph.2021.1604249, PMID: 34675760 PMC8525285

[53] Thomas LM D'Ambruoso L Balabanova D. Use of verbal autopsy and social autopsy in humanitarian crises. BMJ Glob Health. (2018) 3:e000640. doi: 10.1136/bmjgh-2017-000640, PMID: 29736275 PMC5935165

[54] Boerma T Eozenou P Evans D Evans T Kieny MP Wagstaff A. Monitoring progress towards universal health coverage at country and global levels. PLoS Med. (2014) 11:e1001731. doi: 10.1371/journal.pmed.1001731, PMID: 25243899 PMC4171369

[55] World Health Organization. *Universal health coverage (UHC)*. (2021). Available at: https://www.who.int/news-room/fact-sheets/detail/universal-health-coverage-(uhc).

